# “Speedballing” to Severe Rhabdomyolysis and Hemodialysis in a 27-Year-Old Male

**DOI:** 10.7759/cureus.20667

**Published:** 2021-12-24

**Authors:** Mani Maheshwari, Hemanthkumar Athiraman

**Affiliations:** 1 Hospital Medicine, Banner Health, Mesa, USA

**Keywords:** substance recreational use, cryptomarket, darknet, methamphetamine use, illicit fentanyl

## Abstract

With the popularity of smartphones, many drug users buy counterfeit pills online that can lead to serious adverse effects and be detrimental to their health. This case report showcases a young 27-year-old male who smoked and snorted four blue pills called “M30,” which were bought online, resulting in severe rhabdomyolysis and fulminant kidney failure requiring regular hemodialysis.

## Introduction

Cryptomarkets and darknet markets are rapidly growing, increasing the amount and variety of drugs sold [1]. For example, a study on the availability of fentanyl on the darknet done by Matthew Ball et al. showed over 7,000 opioids available and as much as 22 kilograms of fentanyl available in one buy, with a variety of shipping methods from over 100 fentanyl vendors from half a dozen darknets [2]. Fentanyl is a rapid-onset, synthetic opioid about 100 times stronger than morphine, which is now prevalent in the recreational drug markets and often found mixed with heroin, cocaine, oxycodone, hydrocodone, and alprazolam [3]. CDC data show that there were 100,306 drug overdose deaths in the United States this past year, increasing by 28.5% from the year before [4]. Aside from death, this mixture of drugs, with methamphetamines, can wreak havoc on the human body, inhibiting the reuptake of monoamines (serotonin, dopamine, and norepinephrine), causing a spike in sympathetic activity, which results in vasoconstriction, leading to hypoperfusion of the kidneys and muscles, eventually causing acute tubular necrosis and rhabdomyolysis [5]. This case report showcases a 27-year-old male who smoked and snorted four “M30” pills bought online, resulting in severe rhabdomyolysis and renal failure requiring regular hemodialysis. These “M30” pills have recently flooded the United States and are counterfeit drugs made to resemble oxycodone 30 mg pills, laced with fentanyl [6].

## Case presentation

**Figure 1:**
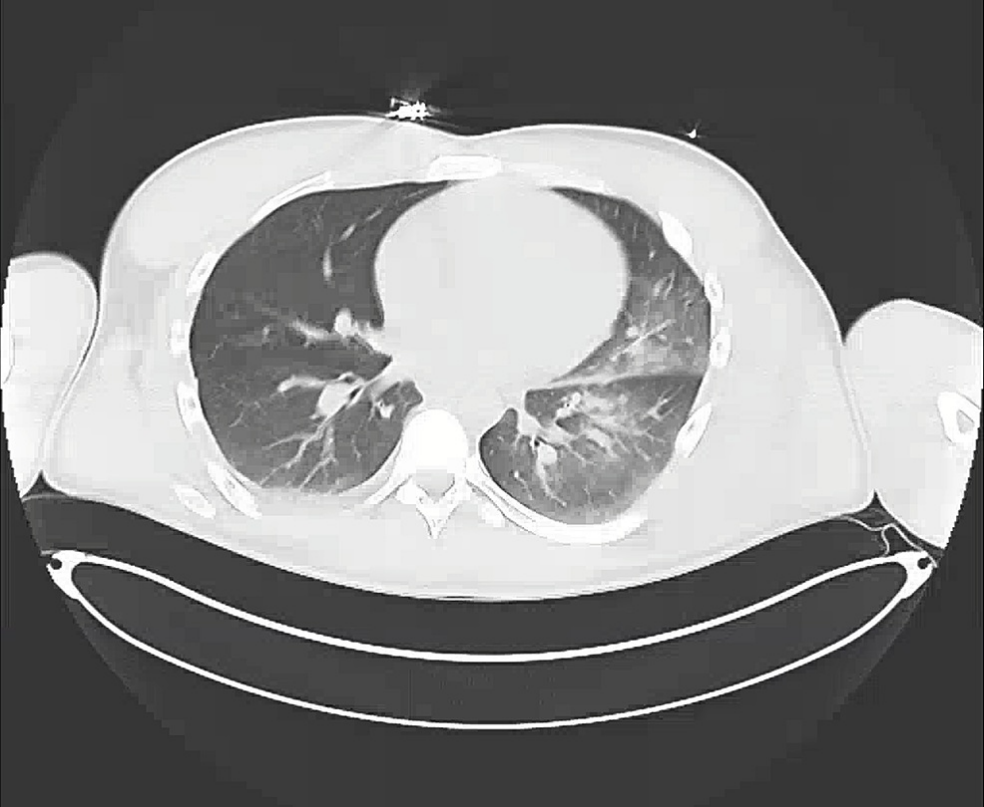
CT of the chest showing bilateral aspiration pneumonia.

A 27-year-old male presented to the ER via ambulance complaining of severe generalized body pain and anxiety after smoking and snorting four unknown blue pills called “M30.” He stated he got these pills online thinking they were oxycodone and had used them many times before, and said, “I don't know what happened this time.” His vital signs were blood pressure level of 89/66, heart rate of 102 beats per minute, respiratory rate of 18 breaths per minute, the temperature of 36.8°C, and 89% oxygen saturation on room air. Laboratory assessment, urinalysis, and urine drug screen results are presented in Table 1. CT of the chest showed bilateral aspiration pneumonia, with left more extensive than right (Figure 1). The patient was confused in the ER, which improved after the administration of Narcan.

**Table 1:** Laboratory assessment, urinalysis, and urine drug screen on admission.

Tests	Values
White blood cell count (K/uL)	27.6 - high
Blood urea nitrogen/creatinine (mg/dL)	22/2.45 - high
Potassium (mmol/L)	6.9 - high
Aspartate aminotransferase (U/L)	1,761 - high
Alanine aminotransferase (U/L)	503 - high
Creatine phosphokinase (U/L)	164,300 - high
Troponin (ng/L)	352 - high
Lactic acid (mmol/L)	4.1 - high
Respiratory procalcitonin (ng/mL)	38.91 - high
Methamphetamine/amphetamine screen, urine	Positive
Cannabinoid (tetrahydrocannabinol) screen, urine	Positive
Fentanyl screen, urine	Positive
Ethanol screen, urine	Positive
Urine color, characteristic	Brown, cloudy
Urine protein (mg/dL)	>=300 (large)
Urine blood (mg/d)	1.00 (large)
Urine nitrite	Positive
Urine RBC	21-50/HPF - high

**Figure 2:**
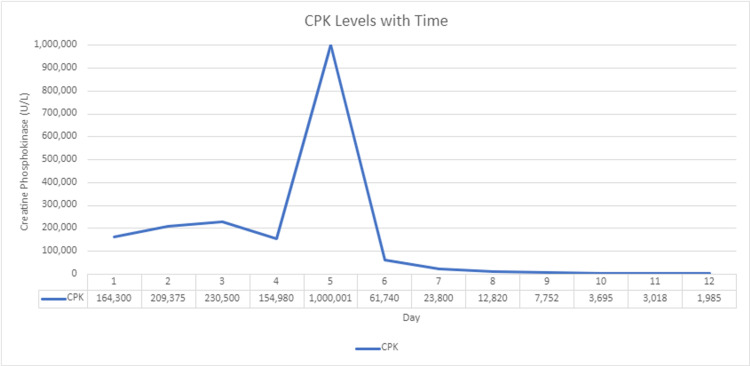
CPK levels with time. CPK, creatine phosphokinase.

The patient was admitted to the telemetry floor with nephrology and toxicology consultations. The patient was started on sodium bicarbonate intravenous fluids, ceftriaxone, azithromycin, sodium zirconium cyclosilicate, and N-acetylcysteine, and lorazepam as needed. Creatine phosphokinase (CPK) levels of the patient continued to rise, reaching >1,000,000 U/L (Figure 2), and the patient developed acute tubular necrosis. Hemodialysis was initiated only on day five, as the patient had repeatedly refused hemodialysis since admission.

**Figure 3:**
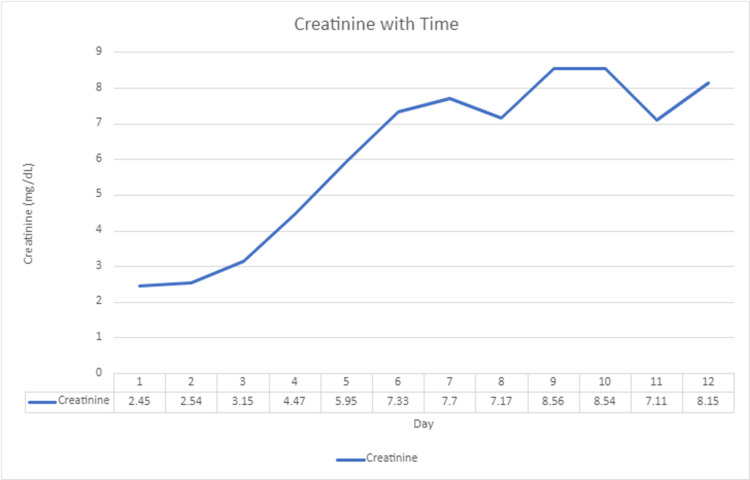
Creatinine with time.

The patient went on to get hemodialysis three times a week since the creatinine continued to climb (Figure 3).

## Discussion

An online survey of over 350 drug users done by Leah Moyle et al. showed that social media and encrypted messaging apps serve as the link coupling buyers and drug suppliers so efficiently that it would not be possible otherwise [7]. The increase in accessibility to drugs lead to a rise in drug overdose deaths to over 100,000 last year, with thousands more affected in dangerous ways.

This patient is an example of severe rhabdomyolysis causing fulminant renal failure, requiring hemodialysis at the young age of 27 years old. He smoked and snorted counterfeit drugs laced with multiple substances, including fentanyl, methamphetamines, and cannabis while ingesting alcohol. Though it is not clear how methamphetamines cause neurotransmitter release, it is known that the primary action of methamphetamines is to increase extracellular levels of dopamine, serotonin, and norepinephrine [8]. This spike in sympathetic activity leads to vasoconstriction, hypoperfusion of kidneys and muscles, resulting in renal failure and muscle breakdown (rhabdomyolysis) [5]. While this is going on, and fentanyl is added to this mix, it slows the activity of the central nervous system and depresses breathing, resulting in the user to feel they have the capacity to use more, which is how the term “speedballing” was created [9]. This creates a fatal or near-fatal storm for the user. This patient suffered severe rhabdomyolysis with CPK levels >1,000,000 U/L, causing acute tubular necrosis and fulminant renal failure requiring regular hemodialysis.

Fentanyl is the deadliest drug in the United States and sits at the core of the opioid crisis [10].

## Conclusions

There is a significant risk of developing severe rhabdomyolysis and renal failure requiring hemodialysis in patients using illicit drugs. Clinicians should be aware of this risk with counterfeit illicit drugs, especially in patients who report using oxycodone blue “M30” pills.
